# Marital status and genetic liability independently predict coronary heart disease incidence

**DOI:** 10.1177/14034948221119634

**Published:** 2022-09-07

**Authors:** Karri Silventoinen, Hannu Lahtinen, Kaarina Korhonen, George Davey Smith, Samuli Ripatti, Tim Morris, Pekka Martikainen

**Affiliations:** 1 Faculty of Social Sciences, University of Helsinki, Finland; 2 Faculty of Medicine, University of Helsinki, Finland; 3 Population Health Sciences, Bristol Medical School, University of Bristol, UK; 4 MRC Integrative Epidemiology Unit, University of Bristol, UK; 5 Institute for Molecular Medicine Finland, Finland; 6 Broad Institute of MIT and Harvard, USA; 7 Centre for Health Equity Studies, Stockholm University, Sweden; 8 Max-Planck-Institute for Demographic Research, Germany

**Keywords:** Marital status, coronary disease, genetics, socioeconomic factors

## Abstract

**Aims:**

Married individuals have a lower coronary heart disease (CHD) risk than non-married, but the mechanisms behind this are not fully understood. We analyzed whether genetic liability to CHD may affect these associations.

**Methods:**

Marital status, a polygenic score of CHD (PGS-CHD), and other risk factors for CHD were measured from 35,444 participants (53% female) in Finnish population-based surveys conducted between 1992 and 2012. During the register-based follow-up until 2020, there were 2439 fatal and non-fatal incident CHD cases. The data were analyzed using linear and Cox regression models.

**Results:**

Divorced and cohabiting men and women had a higher genetic risk of CHD than married individuals, but the difference was very small (0.023–0.058 standard deviation of PGS-CHD, *p*-values 0.011–0.429). Both marital status and PGS-CHD were associated with CHD incidence, but the associations were largely independent. Adjusting for behavioral and metabolic risk factors for CHD explained part of these associations (11–20%). No interaction was found between marital status and PGS-CHD for CHD incidence.

**Conclusions:**

We showed minor differences between the marital status categories in PGS-CHD and demonstrated that marital status and genetic liability predicted CHD incidence largely independently. This emphasizes the need to measure multiple risk factors when predicting CHD risk.

## Introduction

Previous studies have established that coronary heart disease (CHD) incidence is lower among married than non-married individuals [1]. These associations can result from both selection into marital status categories according to CHD risk factors and the effect of marital status on CHD risk, but the direct evidence on either of these mechanisms is still limited [2]. Genetic factors also affect CHD risk [3], and marital status may affect CHD risk by reinforcing or suppressing genetic susceptibility. A previous study found that intimate relationships can suppress the genetic susceptibility to alcohol consumption [4], but little is known about how marital status may interact with genetic susceptibility to CHD. This could explain some of the between-individual variation in the negative health effects of being unmarried or divorced. This, in turn, could have public health implications, as these more susceptible individuals may benefit from targeted health care services.

Here we used genetic data to investigate these relationships. First, we analyzed differences in the genetic liability to CHD as indexed by a polygenic score of CHD (PGS-CHD) between marital status categories. Second, we examined the effect of adjusting for PGS-CHD on marital status differences in CHD incidence. Third, we analyzed possible interaction between marital status and genetic risk when predicting CHD incidence.

## Data and methods

Finnish population-based health surveys conducted between 1992 and 2017 were pooled together; the response rates varied between 65% and 93% [5]. These surveys, including a self-administrated questionnaire and a clinical health examination, were linked to population registers. Baseline marital status was classified as married, unmarried, cohabiting, divorced, and widowed. Analyses were restricted for those between 30 and 70 years of age at baseline.

First, we studied how PGS-CHD was associated with marital status using a linear regression model. PGS-CHD was based on a genome-wide association study of ischemic heart disease in the UK Biobank [6]. Linkage-disequilibrium-weighted scores were calculated with SBayesR using an external linkage disequilibrium matrix for single nucleotide polymorphism (SNP) variants included in HapMap3 with a minor allele frequency of at least 0.01 in our data. PGS-CHD was standardized to have a mean of 0 and standard deviation (SD) of 1. We had 35,444 participants (53% female).

Second, we studied how marital status was associated with CHD incidence and how this association was attenuated or modified by PGS-CHD using Cox proportional hazards models. Non-fatal incident CHD events were based on The Finnish Hospital Discharge Register (ICD-9 codes 410 or 4110 and ICD-10 codes I20.0 and I21–I22) and fatal events on the National Mortality Register (ICD-9 codes 410–414, and 798, excluding 7980A and ICD-10 codes I20–I25, I46, R96, and R98) covering the entire Finnish population. Those who had a CHD event prior to baseline were removed, and those who died from other causes than CHD were censored at the time of death. Cox proportional hazards assumptions were not violated when inspected graphically. Additionally, we calculated population attributable fractions (PAF) to quantify the contribution of all marital status categories on CHD risk. We conducted separate models to adjust the results for (i) PGS-CHD, (ii) education, and (iii) body mass index (BMI, kg/m^2^), regular smoking status, systolic and diastolic blood pressure, total cholesterol, and high-density lipoprotein cholesterol (Supplementary Table I). We observed 2,439 incident CHD cases (33% fatal cases and 32% in females) during the 504,061 person years until the end of follow-up on December 31 2019.

All analyses were adjusted for the first 10 principal components of genomic structure, participant age at baseline, five geographic areas of residence, and a combination of baseline year and genotyping batch dummies. The genetic principal components and PGS-CHD were calculated by PLink 1.9 and GTCB software and statistical models were conducted using Stata 16.

## Results

Cohabiting and divorced men and women had a slightly higher genetic risk of CHD measured as PGS-CHD than those who were married, whereas for unmarried men and women, the difference compared with those married was negligible (Table I). Widowed women had a slightly higher genetic risk, but there were too few widowed men to draw firm conclusions. We did not find evidence of interaction between sex and marital status for PGS-CHD (*p* = 0.16).

**Table I.** Number of participants and regression models predicting standardized polygenic scores for coronary heart disease with 95% confidence intervals by marital status among men and women.^1^

Marital status	Men				Women			
	*N*	β	95% confidence intervals		*N*	β	95% confidence intervals	
			LL	UL			LL	UL
Married	10,552	reference			11,306	reference		
Unmarried	2137	0.004	−0.044	0.051	1980	−0.005	−0.053	0.042
Cohabiting	2356	0.042	−0.005	0.088	2457	0.036	−0.008	0.081
Divorced	1342	0.023	−0.034	0.080	2368	0.058	0.013	0.102
Widowed	171	−0.090	−0.243	0.063	775	0.090	0.016	0.164

Regression coefficients (β) with lower (LL) and upper limits (UL) of 95% confidence intervals.

1 Adjusted for age at baseline, geographic area of residence, 10 genetic principal components, and genotyping batch-baseline year combination.

Both PGS-CHD and marital status were strongly associated with CHD incidence (Table II). For both men and women, married individuals had the lowest CHD risk (Model 1). Adjusting for PGS-CHD attenuated the hazard ratios (HRs) for cohabiting men and women (Model 2). Adjusting for education attenuated marital status differences for men (Model 3), while adjusting for behavioral and metabolic risk factors for CHD attenuated differences both in men and women (Model 4). Adjusting for marital status (Model 2) and education (Model 3) had little effect on the HR of PGS-CHD, but adjusting the results for behavioral and metabolic risk factors for CHD slightly decreased the HR (Model 4). Together, the full adjustment explained 20% of marital status differences in CHD incidence as measured by PAF in men and 16% in women, whereas for PGS-CHD they explained 11% and 15% of the increased risk, respectively. We did not find evidence of interactions between marital status and PGS-CHD in men (*p* = 0.80) or in women (*p* = 0.69), and the interaction terms were weak for all marital status categories.

**Table II.** Hazard ratios of coronary heart disease incidence for standardized polygenic risk score of coronary heart disease and marital status in men and women.

Marital status	Model 1			Model 2			Model 3			Model 4		
	HR	95% confidence intervals		HR	95% confidence intervals		HR	95% confidence intervals		HR	95% confidence intervals	
		LL	UL		LL	UL		LL	UL		LL	UL
Men^1^												
PRS-CHD	1.35	1.28	1.41	1.35	1.28	1.41	1.34	1.27	1.40	1.31	1.25	1.37
Married	1.00			1.00			1.00			1.00		
Unmarried	1.49	1.28	1.73	1.49	1.28	1.74	1.41	1.21	1.64	1.40	1.20	1.64
Cohabiting	1.28	1.08	1.52	1.26	1.06	1.49	1.21	1.02	1.44	1.17	0.98	1.39
Divorced	1.43	1.21	1.68	1.43	1.21	1.68	1.38	1.17	1.63	1.30	1.10	1.53
Widowed	1.18	0.80	1.71	1.23	0.84	1.80	1.22	0.84	1.79	1.18	0.80	1.72
PAF^3^	0.094	0.069	0.119	0.094	0.068	0.119	0.083	0.056	0.110	0.075	0.046	0.102
Women^2^												
PRS-CHD	1.32	1.23	1.42	1.32	1.23	1.42	1.31	1.22	1.40	1.27	1.19	1.37
Married	1.00			1.00			1.00			1.00		
Unmarried	1.15	0.89	1.50	1.17	0.90	1.52	1.24	0.95	1.61	1.25	0.96	1.62
Cohabiting	1.19	0.89	1.58	1.15	0.87	1.54	1.16	0.87	1.54	1.08	0.81	1.43
Divorced	1.50	1.24	1.82	1.49	1.23	1.80	1.49	1.23	1.81	1.38	1.14	1.68
Widowed	1.38	1.08	1.77	1.35	1.06	1.74	1.32	1.03	1.70	1.27	0.99	1.63
PAF^3^	0.112	0.064	0.158	0.112	0.064	0.158	0.111	0.063	0.157	0.094	0.043	0.143

Hazard ratios (HR) with lower (LL) and upper limits (UL) of 95% confidence intervals.

Model 1: Adjusted for age at baseline, geographic area of residence, 10 genetic principal components, and genotyping batch-baseline year combination.

Model 2: Adjusted for Model 1+CHD-PRS or marital status.

Model 3: Adjusted for Model 2+education.

Model 4: Adjusted for Model 3+smoking+BMI+systolic blood pressure+diastolic blood pressure+total cholesterol+high-density lipoprotein cholesterol.

1 PRS*marital status interaction in men when interaction terms are added to Model 2 (χ^2^=1.65 (4); *p* = 0.80 in likelihood ratio test against Model 2): married (reference category), unmarried (1.01; 95% CI 0.87, 1.18), cohabiting (1.00; 95% CI 0.85, 1.18), divorced (1.03; 95% CI 0.88, 1.22), and widowed (1.30; 95% CI 0.85, 2.00).

2 PRS*marital status interaction in women when interaction terms are added to Model 2 (χ^2^=2.25 (4); *p* = 0.69 in likelihood ratio test against Model 2): married (reference category), unmarried (0.84; 95% CI 0.64, 1.08), cohabiting (0.99; 95% CI 0.75, 1.30), divorced (1.01; 95% CI 0.83, 1.22), and widowed (1.04; 95% CI 0.82, 1.33).

3 Population attributable fraction for CHD incidence by marital status.

## Discussion

We found only modest evidence that cohabiting and divorced men and women had higher genetic risk of CHD. The adjustment for PGS-CHD explained a minor part of the excess phenotypic CHD risk of those cohabiting as compared to married, and had essentially no effect on the excess risk of the divorced. The largely independent associations of marital status and PGS-CHD with CHD incidence suggest that they capture different aspects of CHD risk. For example, behavioral risk factors are likely to explain a part of the association between marital status and CHD risk [7], whereas it is possible that PGS-CHD reflects a physiological susceptibility to developing CHD. However, the pathways from genes to CHD risk are complex, and our knowledge is still limited on factors mediating these associations [8].

Both marital status and PGS-CHD were associated with CHD risk. For example, being divorced was associated with increased CHD risk comparable to 1 SD difference of PGS-CHD in both men and women. We did not find evidence that marital status modified the effect of genetic susceptibility on CHD incidence. These results are consistent with recent studies finding little evidence on the multiplicative interactions of genetic susceptibility with lifestyle [9] and socioeconomic factors [10].

The strengths of our data were the large sample size, the high response rates for the baseline surveys, and register-based CHD incidence data minimizing selection bias. We observed a number of behavioral and metabolic risk factors for CHD, but they explained only a minor part of the CHD risk associated with marital status and PGS-CHD. More comprehensive and repeated measures could give more evidence on mediating pathways.

In conclusion, we observed only minor differences between marital status categories in the genetic risk of CHD. Behavioral and metabolic factors, marital status, and genetic liability affect CHD risk largely independently, emphasizing the need to measure multiple risk factors when predicting CHD risk. The risk of CHD is formed by a combination of biological and social risk factors, and thus both aspects should be considered in policies targeted to decrease CHD incidence.

## Supplemental Material

sj-docx-1-sjp-10.1177_14034948221119634 – Supplemental material for Marital status and genetic liability independently predict coronary heart disease incidence Click here for additional data file. Supplemental material, sj-docx-1-sjp-10.1177_14034948221119634 for Marital status and genetic liability independently predict coronary heart disease incidence by Karri Silventoinen, Hannu Lahtinen, Kaarina Korhonen, George Davey Smith, Samuli Ripatti, Tim Morris and Pekka Martikainen in Scandinavian Journal of Public Health
