# Supplementary material for: Marital status and genetic liability independently predict coronary heart disease incidence
Source: Scand J Public Health. 2022 Sep 7;52(1):1–4. doi: 10.1177/14034948221119634 (PMC10845822; doi:10.1177/14034948221119634)
Supplement: sj-docx-1-sjp-10.1177_14034948221119634 – Supplemental material for Marital status and genetic liability independently predict coronary heart disease incidence [file sj-docx-1-sjp-10.1177_14034948221119634.docx]

Supplementary table 1. Distribution of covariates (means and standard deviations or percentages) by marital status categories and sex.^1^

|  | Men | | | | | Women | | | | |
| --- | --- | --- | --- | --- | --- | --- | --- | --- | --- | --- |
|  | Unmarried | Married | Cohabiting | Divorced | Widow | Unmarried | Married | Cohabiting | Divorced | Widow |
| Age at baseline  (years)^2^ | 45 (11) | 51 (11) | 45 (11) | 53 (10) | 62 (8) | 46 (12) | 50 (11) | 44 (11) | 52 (10) | 62 (7) |
| BMI (kg/m^2^)^3^ | 27 (4.9) | 27 (4.0) | 27 (4.0) | 27 (4.4) | 27 (3.7) | 26 (5.3) | 27 (5.1) | 26 (5.1) | 27 (5.4) | 29 (5.5) |
| Systolic blood pressure (mmHg)^4^ | 136 (18) | 137 (18) | 134 (16) | 138 (19) | 145 (20) | 129 (19) | 132 (20) | 127 (18) | 132 (19) | 142 (20) |
| Diastolic blood pressure (mmHg)^4^ | 84 (12) | 84 (11) | 83 (11) | 85 (11) | 86 (11) | 79 (11) | 79 (11) | 78 (10) | 80 (10) | 82 (11) |
| HDL cholesterol  (mmol/l)^5^ | 1.3 (0.4) | 1.3 (0.3) | 1.3 (0.3) | 1.3 (0.4) | 1.3 (0.4) | 1.6 (0.4) | 1.6 (0.4) | 1.6 (0.4) | 1.6 (0.4) | 1.6 (0.4) |
| Total cholesterol  (mmol/l)^5^ | 5.5 (1.1) | 5.6 (1.1) | 5.5 (1.1) | 5.6 (1.2) | 5.6 (1.2) | 5.4 (1.0) | 5.6 (1.1) | 5.3 (1.0) | 5.6 (1.0) | 5.9 (1.1) |
| Education (%)^6^ |  |  |  |  |  |  |  |  |  |  |
| Basic education | 30 | 28 | 24 | 34 | 51 | 18 | 26 | 17 | 31 | 49 |
| Vocational secondary | 38 | 34 | 39 | 35 | 25 | 27 | 30 | 32 | 31 | 29 |
| Academic secondary | 8 | 4 | 8 | 4 | 2 | 10 | 6 | 11 | 6 | 2 |
| Lower tertiary | 16 | 23 | 21 | 21 | 13 | 31 | 27 | 29 | 24 | 17 |
| Higher tertiary | 8 | 11 | 8 | 6 | 9 | 14 | 11 | 11 | 8 | 3 |
| Smoking (%)^6^ |  |  |  |  |  |  |  |  |  |  |
| Never smoker | 46 | 43 | 36 | 29 | 34 | 62 | 66 | 52 | 51 | 68 |
| Former smoker | 16 | 31 | 25 | 25 | 37 | 12 | 18 | 19 | 17 | 16 |
| Current smoker | 38 | 26 | 39 | 46 | 29 | 26 | 16 | 29 | 32 | 16 |

^1^Marital status was assessed by a structured question in the self-administrated baseline questionnaire. The exception was the FINRISK survey in 1992, when the both cohabiting and married were measured as a single category. In this case, marriages were inferred from register-based information. In addition, we used register based information for 46 individuals with missing self-reported marital status.

^2^Age was derived from the Finnish population register.

^3^BMI was calculated from measured body weight (precision to 0.1 kg) and measured height (precision to 0.1 cm) in the baseline health examination when the participants were in light clothing and bare footed.

^4^Systolic and diastolic blood pressures were measured two or three times in the baseline health examination and the means of these measures were used.

^5^HDL and total cholesterol were determined at the Central Laboratory of the Finnish Institute for Health and Welfare from the frozen serum blood sample participants gave in the baseline health examination.

^6^Education and smoking were assessed by structured questions in the self-administrated baseline questionnaire.
